# Phase I trial and pharmacokinetic study of tanibirumab, a fully human monoclonal antibody to vascular endothelial growth factor receptor 2, in patients with refractory solid tumors

**DOI:** 10.1007/s10637-017-0463-y

**Published:** 2017-04-08

**Authors:** Su Jin Lee, Seon Young Lee, Weon Sup Lee, Jin San Yoo, Jong-Mu Sun, Jeeyun Lee, Se Hoon Park, Joon Oh Park, Myung-Ju Ahn, Ho Yeong Lim, Won Ki Kang, Young Suk Park

**Affiliations:** 1 Division of Hematology-Oncology, Department of Medicine, Samsung Medical Center, Sungkyunkwan University School of Medicine, 81 Irwon-ro Gangnam-gu, Seoul, 06351 South Korea; 2 Pharmabcine Inc., Daejeon, South Korea

**Keywords:** VEGFR-2 inhibitor, Tanibirumab, Anti-angiogenic agent, Phase I

## Abstract

*Background* Tanibirumab is a fully human monoclonal antibody to vascular endothelial growth factor receptor 2 (VEGFR-2). We conducted a first-in-human phase I study of tanibirumab in patients with solid tumors refractory to standard chemotherapy. Primary endpoints were evaluating safety, pharmacokinetics (PKs), estimating maximum-tolerated dose (MTD) and recommended phase II dose (RP2D). *Methods* We designed our study to escalate tanibirumab at 9 different dose levels with a 3 + 3 method and tanibirumab (1–28 mg/kg) was administered intravenously on D1, 8, 15 in 28-day courses. Dose limiting toxicities (DLTs) were only assessed during the first cycle of treatment and response evaluation was performed every 2 cycles. The effects of tanibirumab on several angiogenic factors were analyzed. *Results* From October 2011 to September 2013, a total of 26 patients with refractory solid tumors were enrolled. The median age was 58 years (range, 27–75) and 20 patients were male. The most common tumor type was colorectal cancer (*N* = 19) and seven patients had a history of previous bevacizumab treatment. As hemangioma continued to occur, the final dose level, 28 mg/kg, was not performed. DLTs were not found, and the MTD was confirmed to be 24 mg/kg. Hemangioma was observed in 16 patients (61.5%), but all were grade 1–2 and disappeared after discontinuation of the study drug. Among the 18 patients in the efficacy set, no objective response was observed, but 11 patients showed stable disease. PKs were characterized by dose-dependent linear exposure and the mean trough concentrations exceeded biologically relevant target levels at 12 mg/kg and above. Serum VEGF, soluble VEGFR-2, and PlGF increased at the 4 mg/kg dose level and above. *Conclusions* Treatment with tanibirumab showed a tolerable toxicity profile and modest clinical efficacy in patients with refractory solid tumors. A phase II trial of tanibirumab is ongoing now.

**Electronic supplementary material:**

The online version of this article (doi:10.1007/s10637-017-0463-y) contains supplementary material, which is available to authorized users.

## Introduction

Angiogenesis is a complex process that includes endothelial cell proliferation and movement, as well as endothelial cell-mediated degradation of the extracellular matrix. The multi-step process of angiogenesis is essential for cancer progression and metastasis [1]. Vascular endothelial growth factor (VEGF) is a key regulator of angiogenesis and is usually up-regulated in cancers [2]. There are two VEGF receptors responsible for angiogenesis; VEGFR-1, also known as Flt-1, and VEGFR-2, also known as KDR. VEGFR-2 is a major mediator of the mitogenic and angiogenic effects of VEGF signaling [3]. The binding of VEGF to VEGFR-2 triggers auto-phosphorylation and results in activation of downstream pathways, including PI3K-AKT and the RAS-RAF-MEK-MAPK signaling network, which is essential for stimulating the proliferation, migration and survival of cancer and endothelial cells.

Various anti-angiogenic agents have been developed to target the VEGF pathway. Bevacizumab (Avastin, Roche), a humanized monoclonal immunoglobulin G1 antibody targeting circulating VEGF, has been shown to improve clinical outcomes in combination with chemotherapy in various cancer types, such as colorectal, lung and ovarian cancers [4–9]. Small molecular inhibitors, like sorafenib (Nexavar, Bayer) and sunitinib (Sutent, Pfizer), targeting VEGFR2 also showed clinical benefit as single agents for hepatocellular carcinoma or renal cell carcinoma [10–13]. Recently, ramucirumab (Cyramza, Lilly), a monoclonal antibody to VEGFR-2, showed a survival benefit when used solo or in combination with chemotherapy [14, 15].

Tanibirumab is a fully human monoclonal antibody (IgG1) derived from a fully human naïve single chain variable fragment (ScFv) phage library. Tanibirumab selectively binds to VEGFR-2, neutralizes the biological activity of VEGFR-2 and, therefore, blocks angiogenesis and inhibits tumor growth and metastasis. In mouse xenograft and orthotopic models, tanibirumab resulted in robust anti-tumor activity as a single agent, with an efficacious dose range of 0.1–10 mg/kg in colorectal, breast, lung and glioblastoma tumor models [16]. Consequently, we planned this first-in-human phase I study to establish the safety profile and maximum-tolerate dose (MTD) of tanibirumab in patients with advanced solid malignancies. We characterized the pharmacokinetics (PK), pharmacodynamics (PD) effects on serum VEGFR-A, soluble VEGFR-2 and PlGF and preformed a preliminary evaluation of antitumor activity.

## Methods

A prospective, single center, open-label study with dose escalation of tanibirumab was conducted in patients with refractory solid tumors. The study was conducted in accordance with the Declaration of Helsinki. The study protocol was approved by the Institutional Review Board at Samsung Medical Center and registered in clinicaltrial.gov NCT#01660360.

### Patient eligibility

Eligibility criteria for study entry were as follows: histologically confirmed, advanced cancers that failed to respond to at least one prior regimen or for which there is no standard therapy; evaluable disease (measurable or non-measurable based on RECIST criteria v 1.1); age > 20 years; ECOG (Eastern Cooperative Oncology Group) performance status 0–2; life expectancy of greater than 12 weeks; hemoglobin ≥9.0 g/dL, absolute neutrophil count ≥1500/μL, platelets ≥100,000/μL, total bilirubin ≤1.5 times the upper limit of normal, AST/ALT ≤2.5 times the upper limit of normal, serum creatinine ≤1.5 mg/dL, PT-INR ≤1.3 times the upper limit of normal, aPTT ≤1.5 times the upper limit of normal and Bazett’s correction QTc < 450 msec in ECG. Patients were excluded from the study if they had undergone radiotherapy, last chemotherapy, or major surgery within the 4 weeks prior to entering the study, had pleural effusion, ascites or leptomeningeal disease as the only manifestation of the current malignancy, had uncontrolled intercurrent illness, active serious infection, the presence of gastrointestinal perforation, tracheoesophageal fistula, grade IV proteinuria, arterial thromboembolic events, hypersensitivity to CHO cell products or other recombined human or humanized antibodies. Written informed consent was required from all patients.

### Administration and dose escalation

Tanibirumab was administered intravenously to each patient over 60 min on Days 1, 8, 15, every four weeks. We designed the study to escalate tanibirumab at 9 different dose levels from 1 mg/kg to 28 mg/kg with a 3 + 3 method. Three patients were accrued to each dose level. If none of the three patients experienced DLT, the dose was increased in a subsequent group of three patients. If DLT occurred in 1 of the 3 initial patients at a particular dose level, then 3 additional patients were treated at the same dose level, resulting in a total of six patients. If a DLT developed in 2 of 6 patients, then enrollment was stopped at this dose level, which was then defined as the MTD. The preceding dose level was designated as the recommended dose for the phase II study. If two of the first 3 patients experienced DLT, then dose escalation was stopped and de-escalated to an intermediate dose. If fewer than two of the six patients experienced DLTs in the last dose level, then this dose level was recommended for the phase II study. Intra-patient dose escalation was not allowed.

### Dose limiting toxicity (DLT) and maximum tolerated dose (MTD)

A DLT was defined as the occurrence of any of following events during the first cycle of therapy: grade 4 thrombocytopenia, grade 4 neutropenia lasting over 7 days, grade 3 or 4 neutropenia accompanied by temperature ≥ 38 °C, grade 3 or 4 non-hematologic toxicity except for diarrhea, nausea, vomiting that responded to standard supportive care. Safety was assessed every week for the first cycle of treatment. Adverse events were evaluated according to the National Cancer Institute Common Terminology Criteria for Adverse Events, version 4.0. All adverse events were evaluated until 30 days after the last dose of tanibirumab.

### Duration of treatment and follow-up

In the absence of treatment delays due to adverse events, treatment continued for up to 1 year or until one of the following criteria was met: disease progression, intercurrent illness that prevented further administration of treatment, unacceptable adverse events and patient withdrawal. Patients were followed for 6 months after removal from the study or until death, whichever occurred first. Patients removed from the study for unacceptable adverse events were followed until resolution or stabilization of the adverse events.

### Analysis groups

All patients who received any amount of tanibirumab were included in the safety analyses. Patients were considered evaluable for the purposes of dose-escalation decisions and establishing the MTD if they satisfied either of the following criteria: received at least two doses of tanibirumab and completed study assessments through the DLT observation window without experiencing a DLT or experienced a DLT and were withdrawn from the study within the DLT observation window.

Radiological (chest X-ray, computed tomography) studies to assess response were performed after every 2 cycles of therapy until disease progression. Response definitions were according to Response Evaluation Criteria in Solid Tumors (RECIST) 1.1. Progression-free survival (PFS) was defined as the time from the date of treatment initiation to the date of the first documentation of disease progression (by radiologic or clinical) or death. Patients with progression-free status were censored at the last date verifying survival. The Kaplan–Meier method was used to estimate the median values of time-to-event variable and progression-free survival (PFS).

### Pharmacokinetic analysis

PK sampling was conducted at several time points; Cycle 1, first dose, pre-, 30 min, 2, 4, 24, 72 h/ s dose, pre- and 30 min/ third dose, pre, 30 min, 2, 4, 24, 72, 168, 336 h and Cycle 2–13, first dose, pre- and third dose 30 min and EOT visits. The pharmacokinetic (PK) parameter estimates of tanibirumab after the first and third doses in cycle 1 were characterized by non-compartmental methods. The following PK parameter estimates were derived: area under the concentration-time curve (AUC), peak concentration (Cmax), trough concentration (Cmin), clearance (CL), volume of distribution (Vd), and terminal elimination half-lives (initial and final, respectively).

### Biomarker study

Several potential biomarkers of angiogenesis were measured at several time points. The angiogenic factors we evaluated are as follows: soluble VEGFR-1, angiopoietin-1, angiopoietin-2, placental growth factor (PlGF), fibroblast growth factors (FGF), platelet-derived growth factor (PDGF)-AB/BB, thrombospondin-2, granulocyte-colony stimulating factor (G-CSF), hepatocyte growth factor (HGF), soluble Tie-2, soluble VEGFR-2, VEGF-A, leptin, follistatin, platelet endothelial cell adhesion molecule (PECAM)-1, and interleukin (IL)-8.

## Results

### Patient characteristics

Twenty six patients were enrolled from to October 2011 to September 2013. The baseline demographics for all patients are shown in Table 1. The median age was 58 years (range, 27–75) and 20 patients (76.9%) were male. All patients except one (*N* = 25, 96.1%) had good performance status (ECOG PS 0–1). The most common tumor type was rectal cancer (*N* = 11), followed by colon cancer (*N* = 8), head and neck squamous cell carcinoma (*N* = 2) and patients with gastric cancer, non-small cell lung cancer, esophageal cancer, adenoid cystic carcinoma, malignant fibrous histiocytoma were enrolled one by one. Seven patients had a history of previous bevacizumab treatment.

**Table 1:** Patient characteristics (*N* = 26)

	No. of patients (%)
Sex	
Male	20 (76.9)
Female	6 (23.1)
Median age (range), years	58 (range, 27–75)
ECOG performance status	
0	4 (15.4)
1	21 (80.8)
2	1 (3.9)
Primary malignancy	
Rectal cancer	11 (42.3)
Colon cancer	8 (30.8)
Head and neck squamous cell carcinoma	2 (7.7)
Gastric cancer	1 (3.9)
Non-small cell lung cancer	1 (3.9)
Esophageal cancer	1 (3.9)
Adenoid cystic carcinoma	1 (3.9)
Malignant fibrous histiocytoma	1 (3.9)
Current site of metastasis	
Lung	24(92.3)
Distant LN	10(38.5)
Liver	10(38.5)
Bone	5(19.2)
Peritoneal seeding	1(3.8)
Pleural seeding	1(3.8)
Prior systemic regimens for metastatic disease	
1	1(3.8)
≥ 2	25(96.2)
Duration of previous bevacizumab treatment (*N* = 7)	23 weeks (range, 6–29)

### Dose escalation and DLT

**Fig. 1:**
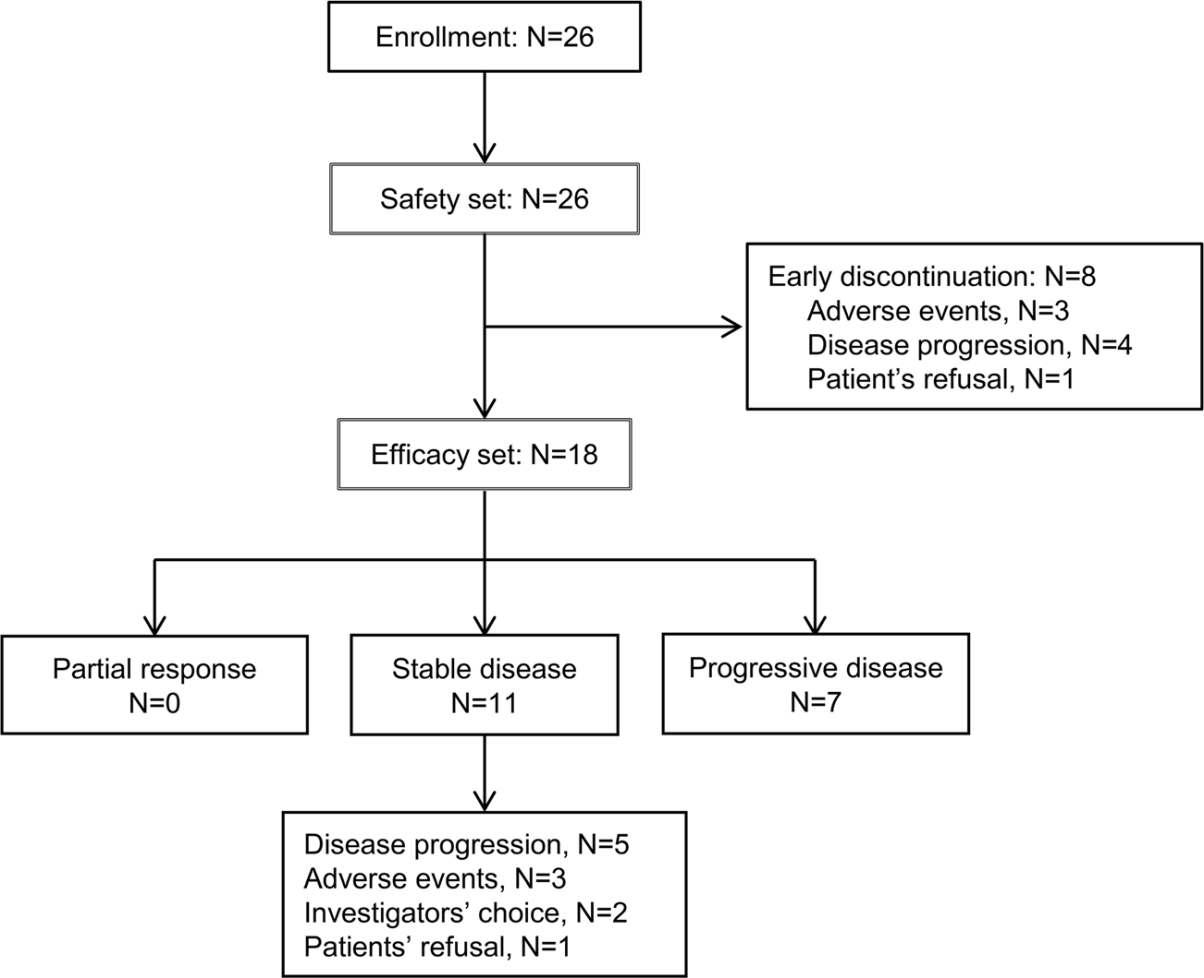
CONSORT diagram of patient enrollment

Patient enrollment and treatment results are shown in Fig. 1. DLT was assessed in 24 patients as two patients were withdrawn before completion of the first cycle of treatment due to ileus and foot fracture, respectively. There was no DLT in a total of 8 dose levels. Even though we planned 9 dose levels extending up to 28 mg/kg, we stopped dose escalation at 24 mg/kg. This was due to the pharmacokinetic results which showed that mean trough concentrations exceeded the biologically relevant target levels at 12 mg/kg and above, and because several hemangiomas occurred. Consequently, the MTD of tanibirumab was established to be 24 mg/kg.

### Overall safety and tolerability

All adverse events in all cycles are shown in Table 2. The most frequently reported drug-related adverse events were hemangioma (*N* = 15, 57.7%), followed by skin rash (*N* = 8, 30.8%), itching (*N* = 5, 19.2%) and fatigue (*N* = 4, 15.4%). Severe adverse events of grade 3 or 4 were observed in two patients (7.7%), irrespective of their potential drug-related attribution. These were neutropenia and spine pathologic compression fracture. We observed hemangioma from dose level 2 (2 mg/kg), usually at the end of cycle 1. Among 16 patients with hemangioma, seven progressed to grade 2 hemangiomas and their treatment was based on a physicians’ decision; temporally holding of tanibirumab, excision of hemangioma or observation. Six cases were pathologically confirmed through biopsy. All hemangioma lesions regressed at 1–2 months after discontinuation of tanibirumab.

**Table 2:** Toxicity profile

Toxicity	Dose level 1–2 (*n* = 6)	Dose level 3–4 (*n* = 6)	Dose level 5–6 (*n* = 7)	Dose level 7–8 (*n* = 7)	Total (*n* = 26)	
Grade	1 2 3 4	1 2 3 4	1 2 3 4	1 2 3 4	All grade (%)	Grade 3/4 (%)
Hematological						
Neutropenia				1 1	1 (3.8)	1 (3.8)
Anemia		1			1 (3.8)	
Thrombocytopenia						
Non-hematological						
Anorexia	1		1	1	3 (11.5)	
Nausea		1		1	2 (7.7)	
Vomiting	2	1			3 (11.5)	
Diarrhea	2				2 (7.7)	
Mucositis		2		1	3 (11.5)	
Myalgia	2			2	4 (15.4)	
Fatigue	1	1 1		2	5 (19.2)	
General weakness	1		2		3 (11.5)	
Headache	1			1	2 (7.7)	
Dizziness		1		1	2 (7.7)	
Skin rash	1	4	2	2	9 (34.6)	
Itching sense	2	2	2		6 (23.1)	
Hemangioma	3	2 2	2 1	2 4	16 (61.5)	
Edema	1		1	1	3 (11.5)	
Hypersensitivity			1 1		2 (7.7)	
Liver enzyme elevation			1		1 (3.8)	

### Immunogenicity

Antibodies to tanibirumab were observed in four samples but these had no neutralizing activity and the plasma concentrations of tanibirumab were unaffected.

### Pharmacokinetic analysis

**Fig. 2:**
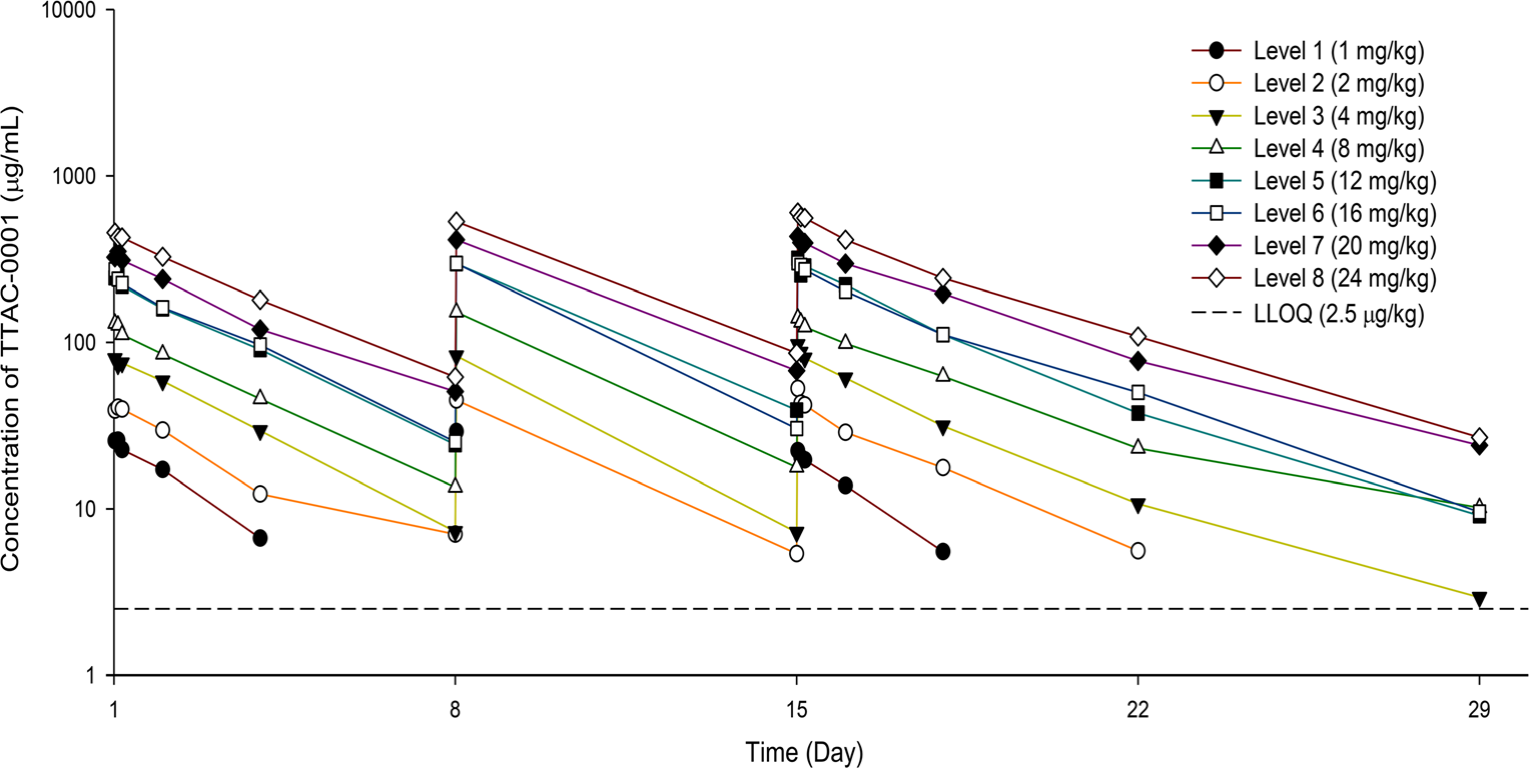
Mean serum concentration time profiles for tanibirumab

Individual PK parameters, including mean trough and other AUC parameters, were obtained from 26 patients (Supplementary Table 1). C_max_ and AUC_inf_ increased proportionately as the dose increased (Fig. 2). Half-lives were 1.6–2.3 days and 1.5–3.0 days at first dose and third dose, respectively, and the increases of C_max_ and AUC did not affect the half-life. As a result, PKs were characterized by dose-dependent linear exposure and mean trough concentrations exceeded biologically relevant target levels at 12 mg/kg and above.

### Biomarker study

**Fig. 3:**
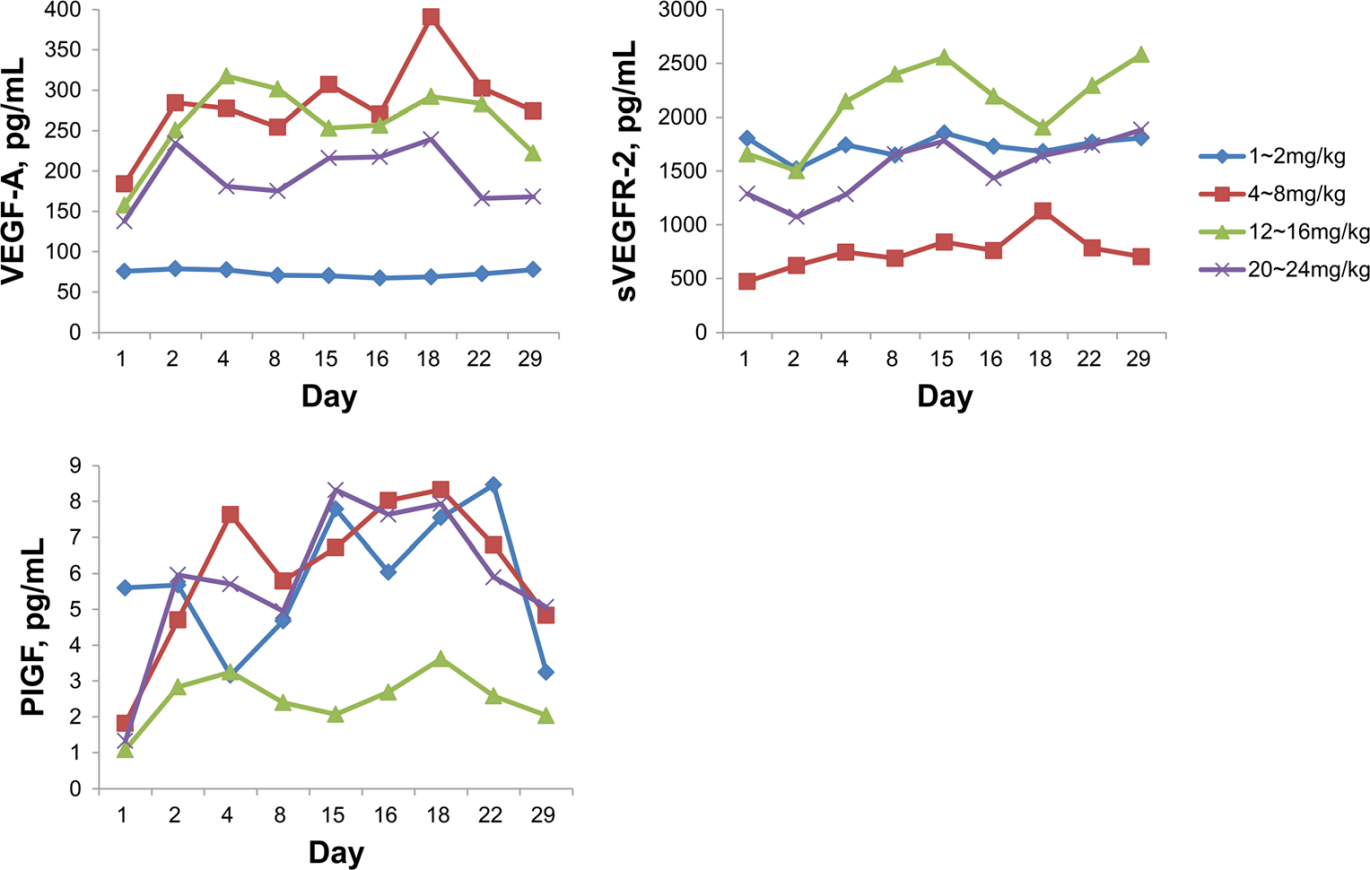
Pharmacodynamic study, scatterplots of VEGF-A, soluble VEGFR-2 and PIGF of raw data from pretreatment values over time after infusion of tanibirumab

Among several factors, serum VEGF, soluble VEGFR-2, and PlGF changed at the 4 mg/kg dose level and above (Fig. 3).

### Efficacy and follow-up details

**Fig. 4:**
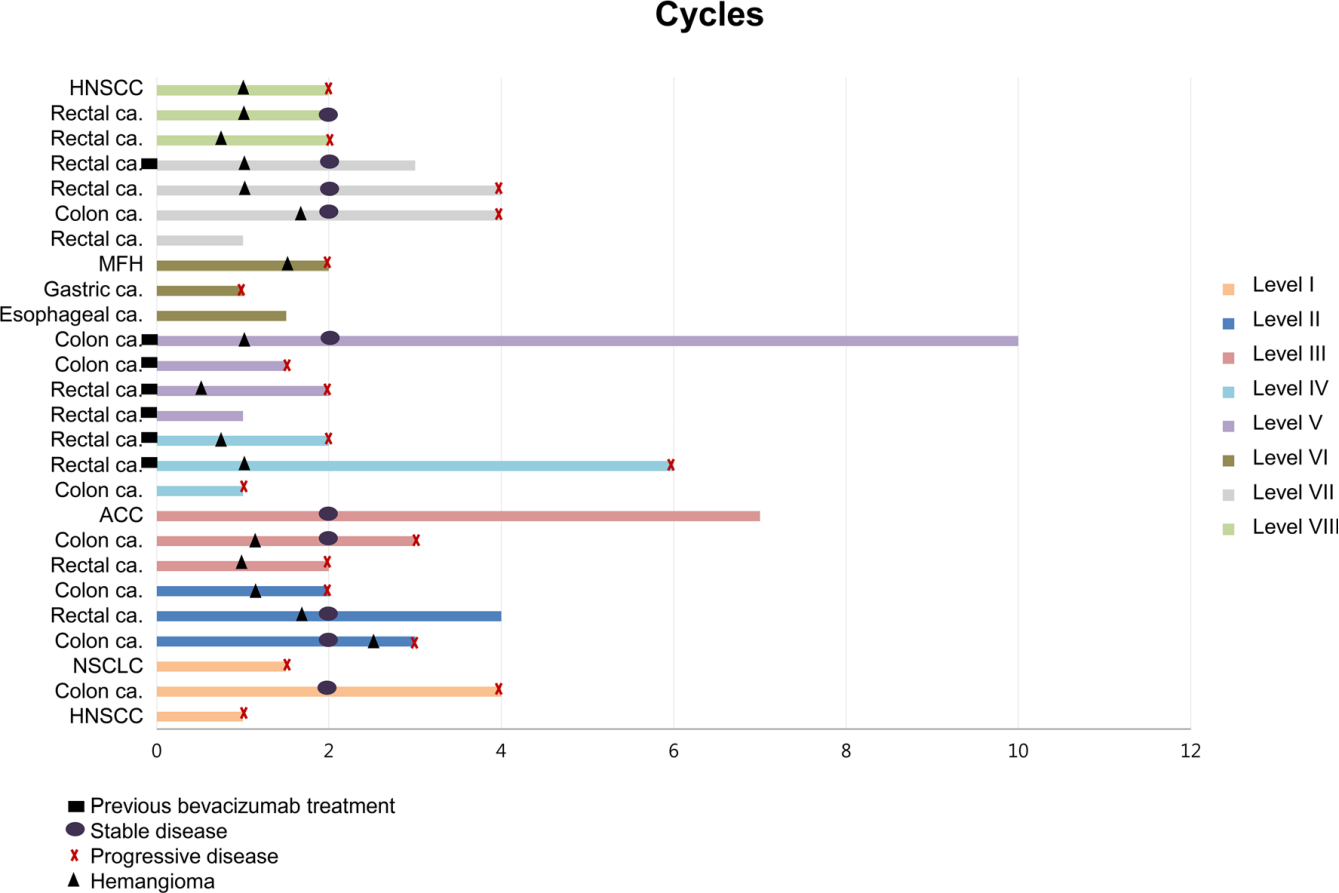
Swimmerplot of individual patient data

In total, 75 cycles were administered with a median of two cycles (range, 1 ~ 10). All patients who completed at least two cycles of therapy were considered evaluable for treatment response (*N* = 18). Among the 18 patients in the efficacy set, no objective response was observed, however, 11 patients showed stable disease with a disease control rate of 61.1% (95% C.I: 38.6–83.6%). Three patients had stable disease for more than 6 months, including patients with adenoid cystic carcinoma, rectal cancer and colon cancer. The median progression-free survival was 3.0 months (95% C.I: 1.9–4.0). Brief Individual data of patients were given on Fig. 4.

## Discussion

Tanibirumab was developed to inhibit angiogenesis through binding to VEGFR2 with high affinity. Tanibirumab, a fully human monoclonal antibody, selectively binds to VEGFR2 and neutralizes the biological activity of VEGFR2. It therefore blocks angiogenesis and inhibits tumor growth and metastasis. Whereas bevacizumab specifically binds to VEGF-A only, tanibirumab blocks all known VEGFs from binding to VEGFR2, which might result in improved clinical efficacy.

In the present study, tanibirumab was well tolerated as a weekly infusion. Even though we initially planned 9 dose levels up to 28 mg/kg, we stopped dose escalation at 24 mg/kg. This was due to the pharmacokinetic results which showed that mean trough concentrations exceeded biologically relevant target levels at 12 mg/kg and above, and due to several occurrences of hemangioma. Target dose (C_trough_ ≥ 20 μg/ml) was previously determined in in vitro HUVEC and in vivo COLO205 model studies. Consequently, the MTD of tanibirumab was determined to be 24 mg/kg.

The tanibirumab PK profile was characterized by dose-proportional elimination and linear exposure. In the PD analysis, serum VEGF-A, soluble VEGFR-2, and PlGF changed at the 4 mg/kg dose level and above. This effect did not seem to be dose related. The increased VEGF-A concentration is likely due to displacement of the receptor-bound natural VEGF-A ligand after tanibirumab treatment. Moreover, this may be a result of hypoxia due to inhibition of the VEGF/VEGFR signaling pathway. Similar results were previously reported in other VEGF/VEGFR inhibitor studies [17–20], and VEGF-A elevation may be a useful marker for VEGFR2 blockade. The concentration of sVEGFR2 was dropped right after tanibirumab injection and restored original level or increased somewhat. It is similar tendency to that of ramucirumab [17]. Binding of tanibirumab to sVEGFR2 may delay the washout of sVEGFR2, which results in increment of sVEGFR2.

Several patients developed hemangiomas in our study. Hemangiomas are formed by an abnormal collection of blood vessels that may resemble tumors. They are usually found on the skin or internal organs and can lead to disfigurement or life-threatening consequences [21, 22]. In our study, all hemangioma events were grade 1 or 2 and resolved upon cessation of tanibirumab administration. Although one patient who had a hemangioma near a colostomy site was withdrawn from the study due to the risk of bleeding, it did not found in the internal organs and the hemangiomas in other patients did not result in discontinuation of tanibirumab. The mechanism through which hemangiomas develop is unclear, however, in the case of infantile hemangioma, hypoxia, estrogen and VEGFR1/VEGFR2 expression have been suggested as possible causes [23, 24]. Hemangioma events were previously reported in other VEGFR2 antibody studies. In a ramucirumab study, one patient receiving 4 mg/kg ramucirumab treatment developed a hemangioma [25]. In a phase I study of CDP-791, a PEGylated di-Fab’ conjugate that binds VEGFR2, seven of 31 patients developed hemangiomas [26]. The biopsy and immunohistochemical staining of these hemangiomas showed that VEGFR2 was widely expressed, but PEG was only present in the parts of the sections that did not bind the anti-phospho-VEGFR2 antibody, suggesting that the drug was biologically active and thereby inhibited receptor activation and phosphorylation. In that study, the mechanism of hemangioma was also inconclusive. However, we can hypothesize about the involvement of two different pathways: the first being the VEGFR2 pathway and the second being other angiogenic factors. Even though signaling through VEGFR2 bound to tanibirumab is inhibited, free VEGFR2 unbound to tanibirumab can be internalized into cells and phosphorylated. This VEGFR2 signaling can induce hemangioma when combined with certain local circumstances [23]. An additional possible explanation is that local hypoxia induced by tanibirumab may induce many kinds of different angiogenic factors, which might ultimately result in hemangioma. To further investigate hemangioma events, biopsy and immunohistochemical staining of many additional angiogenic factors will be needed.

In summary, the anti-VEGFR2 antibody tanibirumab was well tolerated and showed signs of clinical activity in multiple solid tumors. Based on the pharmacokinetic/safety profiles of tanibirumab, a phase II study could employ dosing schedules of 12 mg/kg - 24 mg/kg every week or 20 mg/kg - 24 mg/kg every two weeks. Similar to other anti-VEGFR/VEGFR agents, predictive biomarkers of clinical benefit have not been identified and should be further investigated.

## Electronic supplementary material

Supplementary table 1 (DOCX 23 kb)
