# Supplementary material for: Phase I trial and pharmacokinetic study of tanibirumab, a fully human monoclonal antibody to vascular endothelial growth factor receptor 2, in patients with refractory solid tumors
Source: Invest New Drugs. 2017 Apr 8;35(6):782–90. doi: 10.1007/s10637-017-0463-y (PMC5694508; doi:10.1007/s10637-017-0463-y)
Supplement: Supplementary file 1 — (DOCX 23 kb) [file 10637_2017_463_MOESM1_ESM.docx]

**Supplementary Table 1** Pharmacokinetics result

| Dose level | Mean | T_1/2_^a^ (day) | | | T_max_ (day) | | | C_max_ (µg/mL) | | | | C_min_ (µg/mL) | | | AUC_last_ (day*µg/mL) | | | AUC_inf_ (day*µg/mL) | | Vd | | CL | | |
| --- | --- | --- | --- | --- | --- | --- | --- | --- | --- | --- | --- | --- | --- | --- | --- | --- | --- | --- | --- | --- | --- | --- | --- | --- |
|  |  | Frequency | | | Frequency | | | Frequency | | | | Frequency | | | Frequency | | | Frequency | | Frequency | | Frequency | | |
|  |  | 1 | 3 | | 1 | 3 | | 1 | 3 | | | 1 | 1 | | 1 (AUC_1-4day_) | | 3  (AUC_15-18day_) | 1 | 3 | 1 | 3 | 1 | | 3 |
| Level 1 | Geometric Mean | 1.6 | 1.5 | | 0.052 | 0.021 | | 26.3 | 22.3 | | | NA | NA | | 42.7 | | 35.1 | 59.6 | 47.5 | 38.91 | 46.83 | 16.78 | | 21.04 |
| (1 mg/kg) | CV (%) | 33.7 | 12.3 | | 94.7 | 0.0 | | 2.6 | 17.2 | | | NA | NA | | 8.1 | | 18.6 | 25.1 | 24.2 | 8.29 | 12.47 | 25.07 | | 24.18 |
| Level 2 | Geometric Mean | 1.7 | 2.5 | | 0.105 | 0.033 | | 41.1 | 53.6 | | | NA | NA | | 74.4 | | 82.1 | 104.2 | 149.4 | 45.92 | 48.38 | 19.20 | | 13.39 |
| (2 mg/kg) | CV (%) | 8.8 | 46.5 | | 41.7 | 94.7 | | 26.2 | 26.5 | | | NA | NA | | 41.8 | | 38.9 | 46.1 | 66.2 | 36.67 | 31.27 | 46.09 | | 66.19 |
| Level 3 | Geometric Mean | 2.1 | 2.2 | | 0.042 | 0.021 | | 84.5 | 96.9 | | | 7.3 | 7.2 | | 152.1 | | 161.9 | 240.6 | 266.0 | 49.25 | 47.02 | 16.62 | | 15.04 |
| (4 mg/kg) | CV (%) | 18.2 | 36.5 | | 179.6 | 0.0 | | 27.3 | 11.6 | | | 29.5 | 49.7 | | 19.2 | | 14.4 | 10.2 | 27.3 | 27.33 | 19.15 | 10.25 | | 27.33 |
| Level 4 | Geometric Mean | 2.2 | 2.8 | | 0.033 | 0.042 | | 131.3 | 145.1 | | | 13.5 | 17.7 | | 227.2 | | 272.5 | 374.4 | 536.0 | 68.57 | 59.80 | 21.37 | | 14.93 |
| (8 mg/kg) | CV (%) | 9.8 | 43.4 | | 94.7 | 179.6 | | 22.7 | 12.9 | | | 59.4 | 67.5 | | 21.8 | | 22.5 | 27.8 | 53.5 | 18.35 | 13.81 | 27.75 | | 53.45 |
| Level 5 | Geometric Mean | 2.3 | 2.2 | | 0.042 | 0.021 | | 281.1 | 318.7 | | | 24.3 | 39.2 | | 439.1 | | 576.4 | 742.0 | 960.1 | 53.75 | 39.54 | 16.17 | | 12.50 |
| (12 mg/kg) | CV (%) | 17.5 | 42.5 | | 94.7 | 0.0 | | 32.7 | 20.7 | | | 20.2 | 18.2 | | 15.9 | | 21.8 | 9.4 | 42.3 | 21.73 | 17.54 | 9.44 | | 42.26 |
| Level 6 | Geometric Mean | 2.3 | 2.2 | | 0.021 | 0.033 | | 271.7 | 308.6 | | | 25.1 | 30.2 | | 449.9 | | 546.9 | 769.9 | 899.6 | 70.05 | 56.00 | 20.78 | | 17.79 |
| (16 mg/kg) | CV (%) | 7.4 | | 17.7 | 0.0 | 94.7 | 25.7 | | | 26.1 | 16.5 | | 47.2 | 21.8 | | 15.5 | | 26.7 | 22.0 | 19.72 | 16.26 | | 26.70 | 22.03 |
| Level 7 | Geometric Mean | 2.0 | | 3.0 | 0.059 | 0.021 | 360.6 | | | 432.3 | 50.6 | | 67.5 | 625.5 | | 835.8 | | 980.2 | 1689.9 | 60.17 | 51.15 | | 20.40 | 11.84 |
| (20 mg/kg) | CV (%) | 4.8 | | 27.0 | 78.5 | 0.0 | 14.7 | | | 12.5 | 32.0 | | 17.8 | 12.9 | | 18.4 | | 13.1 | 35.9 | 13.63 | 12.93 | | 13.08 | 35.93 |
| Level 8 | Geometric Mean | 2.3 | | 2.4 | 0.042 | 0.021 | 464.7 | | | 601.8 | 61.8 | | 86.2 | 870.5 | | 1131.2 | | 1471.1 | 1964.0 | 54.16 | 42.27 | | 16.31 | 12.22 |
| (24 mg/kg) | CV (%) | 15.0 | | 10.2 | 179.6 | 0.0 | 13.3 | | | 8.8 | 10.7 | | 15.9 | 26.0 | | 11.7 | | 16.8 | 11.4 | 31.82 | 13.61 | | 16.79 | 11.35 |
